# ‘I wouldn’t want one or the other’: Understanding parents’ preferences for direct support or parent coaching for young autistic children

**DOI:** 10.1177/13623613241287300

**Published:** 2024-10-12

**Authors:** Phoebe Jordan, Carla Wallace-Watkin, Jessica Tupou, Sarah Pillar, Hannah Waddington

**Affiliations:** 1 Te Herenga Waka – Victoria University of Wellington, New Zealand; 2 The University of Western Australia, Australia

**Keywords:** autism, direct support, low-intensity support, parent coaching, template analysis

## Abstract

There has been considerable research into the barriers and facilitators of coaching parents of autistic children. However, little is known about parents’ preferences for this approach *compared* to support provided directly by a clinician to an autistic or potentially autistic child. This study aimed to examine parents’ relative preferences for parent coaching and direct clinician support. A total of 22 families who had received both approaches for their autistic child quantitatively indicated which they preferred. We then used semi-structured interviews with 11 of these participating parents to further investigate the reasons underlying these preferences. We used multiple methods to ensure trustworthiness and credibility. Four themes were identified through template analysis: (1) ‘I wouldn’t want one without the other’, (2) ‘It forced me outside my comfort zone’, (3) ‘It’s just about different types of learners’ and (4) ‘If our child is happy, then we are happy’. Overall, results indicated that parents appreciated characteristics of both approaches and felt that they complimented each other in helping themselves and their child. However, when forced to choose, parents generally expressed a preference for direct support. Many parents discussed their belief that their child experienced more enjoyment and progressed further through direct support. These findings emphasise the importance of honouring families’ preferences in the delivery of supports.

**Lay abstract:**

Professionals often support autistic children by working with them directly (direct support) or by coaching their parents. We know a lot about what parents think about parent coaching, but we do not know as much about what they think about direct support. We also do not know whether parents prefer parent coaching or direct support. The current study involved 22 parents who each received 2 h a week of direct support for their autistic child and up to 1 h a week of parent coaching for 6 months. At the end of 6 months, all these parents indicated in a survey whether they preferred parent coaching or direct support. Eleven of these participating parents also chose to take part in an interview to understand more about these preferences. Our findings suggest that parents generally liked both supports and believed they worked well together; however, they preferred direct support over parent coaching. While parents think that both approaches are beneficial, there are strengths and challenges of each. These findings emphasise the importance of parent choice in the delivery of support. It may also be possible to adapt both approaches to address some of the identified challenges and improve the whole family’s experience.

Autism is a neurodevelopmental condition that can involve differences in social communication and specialised interests (DeFilippis & Wagner, 2016). Autism is a form of neurodivergence, meaning that autistic people have brain-based differences, for example, in how they process and communicate information (Chapman & Botha, 2022; den Houting, 2019). The neurodiversity movement encourages using neurodiversity-affirming approaches to support neurodivergent people (den Houting, 2019). This includes identifying each autistic individual’s unique strengths, challenges and support needs (Lord et al., 2020). Approximately 1 in 36 children are currently diagnosed as autistic in the United States (Maenner et al., 2023). As families and communities learn more about autism and how to help autistic children, the demand for accessible early support is also growing (Lord et al., 2018).

One method of support for autistic children is parent coaching. Parent coaching is a broad term encompassing many strategies that a coach uses to help a parent to support their autistic child (Bearss et al., 2015; Malucelli et al., 2021). Parents are ideally situated to implement strategies with their children across different situations and environments (Nevill et al., 2016). This means children can benefit from parental strategy use throughout the day and in different situations, which can increase generalisation and maintenance (Ryberg, 2015). Furthermore, coaching allows parents to be part of their child’s support rather than being a bystander (Rogers et al., 2018). Teaching parents to support their child can help enhance their own confidence and feeling of competence and decrease their stress levels (Jurek et al., 2022; Rogers et al., 2018). Through coaching, parents can generally learn to implement supports with a reasonable degree of accuracy (Penney & Schwartz, 2018).

There has been a significant amount of research examining parents’ perceptions of parent coaching. Many parents feel like their relationships with their children benefit from them being part of their child’s support (Stahmer et al., 2016). Parents have also described feelings of empowerment that have developed from experiencing parent coaching (Jurek et al., 2022). However, parents have also reported challenges associated with parent coaching programmes, such as feeling overwhelmed or not having time to implement the techniques (Freuler et al., 2013; Jurek et al., 2022). One challenge parents may encounter is that their child’s mood, behaviour or illness impact implementation, making it difficult for parents to deliver strategies consistently (Abouzeid et al., 2020). Also, some parents may have difficulty finding time to deliver the support to their children and may not be able to integrate strategies into their daily routines (Stahmer et al., 2016). These findings suggest that while parents generally find coaching beneficial, it is not without its inherent challenges.

One-on-one direct support from a clinician is another common support option for autistic children (Hillman, 2018). One-on-one direct support refers to a trained clinician working directly with the autistic child, rather than upskilling the parent. There has been significant quantitative research into the effects of one-on-one direct support, which has been associated with positive outcomes across different approaches, including developmental, behavioural and naturalistic developmental behaviour supports (Sandbank et al., 2023). Sandbank et al. (2023) also note that many studies included in their review had a high risk of bias, meaning these effects should be interpreted with caution. However, there is limited qualitative research regarding perceptions, including parent perceptions of direct clinician support across approaches. The existing research, albeit limited, indicates that parents find the direct approach valid, effective and socially meaningful (Bent et al., 2023; Ogilvie & McCrudden, 2017). For example, parents of autistic children participating in a university-affiliated one-on-one and group-based direct clinician support indicated that they saw considerable gains in their children who had accessed the service, which they attributed to the expertise of the staff (Bent et al., 2023). However, these parents reported negative experiences when their child’s time in the programme ended. Previous studies have also highlighted the importance of the relationships between clinician and parent as well as clinician and child. Indeed, parents have reported relationships to be a strong factor in the perceived success of one-on-one direct support (Ogilvie & McCrudden, 2017; Seo et al., 2022). The positive aspects of direct support may indicate that it should be offered as an option for families, instead of relying solely on parent coaching (Wallace-Watkin et al., 2020). The challenges of direct support appear to differ from those of parent coaching. For example, parents have reported difficulties with the structure of, and ability to observe, direct support and doubts about whether their child’s progress was due to the support or natural child development (Seo et al., 2022).

It appears that only one study has compared parent perceptions of parent coaching to direct support. Seo et al. (2022) evaluated the perceptions and experiences of four parents of autistic children who participated in one 1-h parent coaching session for 12 weeks and then two 1-h direct support sessions for 12 weeks. Both the parent coaching and support phases were followed by a semi-structured interview, which predominantly focused on parents’ perceptions of each approach in isolation. However, findings from the few questions related to comparing approaches suggested parents preferred the therapists’ approach to implementing the techniques and believed the therapist had a greater impact on their child. However, parents preferred parent coaching overall because they were able to be more involved and felt more empowered.

Providers must ensure they deliver supports that fulfil the needs of children and families (Klatte et al., 2023). This includes understanding parents’ comparative preferences for supports and the reasons behind these preferences. This is because parents are often the key decision-makers for young children, and the provision of support is finite, both in terms of family time and available funding (Kasilingam et al., 2019). Parents are unlikely to continue a support that they think is too expensive, stressful, time-consuming or not suited to their child and family (Jurek et al., 2022; Wallace-Watkin et al., 2022).

The current study took place in New Zealand, a country with relatively low resourcing for autism support (Kasilingam et al., 2019; van der Meer et al., 2023). Parent coaching is the primary model of service delivery in New Zealand, and direct provision of naturalistic developmental behavioural support, for example, is not funded by the government (Wallace-Watkin et al., 2022). Indeed, a large proportion of parents report an unmet demand for direct delivery of support for their autistic child (Wallace-Watkin et al., 2022). Given this predominant reliance on parent coaching and parental demand for direct support, this research aimed to understand parents’ perceptions of, and preferences for, these two approaches to supporting their autistic child. The study involved asking parents of children who were either autistic or showing signs of autism, and who had recently participated in a 6-month low-intensity programme of direct support and parent coaching, about their preferred approach. We then conducted semi-structured interviews with a proportion of these parents to further understand the reasons behind these preferences.

## Method

### Participants and setting

Participants were recruited from a larger randomised controlled trial (RCT) evaluating the effectiveness of low-intensity Early Start Denver Model (ESDM) direct support and parent coaching (Waddington et al., 2023). In the broader RCT, families were randomly allocated to the low-intensity support group or the community group. Participants in the support group received parent coaching and direct therapy, and those in the community group received monthly phone calls to provide general, non-therapeutic support and assistance with referrals. Participants were eligible to participate in this RCT if their child was between 1 and 4½ years old; was autistic or potentially autistic (referred to herein as ‘autistic’); had no other serious medical, genetic or neurological conditions; was born after 34 weeks; was not receiving more than 15 h per week of early intervention; had not received more than one ESDM direct support or parent coaching session or more than two ESDM workshops; and was able to attend sessions in the Wellington region. Parents in the current study were recruited from the broader RCT through a purposive sampling strategy. This involved identifying all parents who were part of the low-intensity support group and had completed the full 6-month duration of the programme. These parents were chosen to ensure that participants had a comprehensive experience of the support programme, which would allow for more informed feedback on their preferences. Out of this selected group, all 22 eligible parents participated by responding to a quantitative survey question regarding their preference for direct support versus parent coaching. This included one parent whose child was diagnosed with a genetic condition during the research, making them ineligible for inclusion in the broader RCT. These 22 parents were also approached to participate in a semi-structured interview aimed at gaining a deeper understanding of their preferences. Out of these, 11 parents indicated that they were interested and consented to participate. Demographic characteristics for all 22 children and parents participating in the research and the subset of 11 children and families are presented in Table 1. Table 2 shows the additional number, amount and type of services accessed by all participants and those who participated in the interviews. The mean number of services accessed by all 22 participants was 1.27, with the most commonly accessed services being speech-language therapy and Wellington Early Intervention Trust.

**Table 1.** Demographic characteristics for participating parents (*n* = 22) and their children (*n* = 22).

Demographic characteristics	All (*n* = 22)	Participated in the interview (*n* = 11)
	*n* (%)	*n* (%)
**Parent characteristics**		
Primary participant		
Mother	17 (77%)	9 (82%)
Father	5 (23%)	2 (18%)
Education		
High school	5 (23%)	4 (36%)
Certificate/trade	4 (18%)	1 (9%)
Under-graduate	8 (36%)	4 (36%)
Post-graduate	5 (23%)	2 (18%)
Employment		
Not employed	10 (46%)	5 (46%)
Part-time	6 (27%)	4 (36%)
Full-time	6 (27%)	2 (18%)
Family income		
$10,001–$60,000	3 (14%)	2 (18%)
$60,001–$80,000	5 (23%)	2 (18%)
$80,001–$100,000	4 (18%)	2 (18%)
$100,001–$150,000	3 (14%)	2 (18%)
$150,001+	4 (18%)	2 (18%)
Prefer not to say	3 (14%)	1 (9%)
**Child characteristics**		
Sex		
Female	7 (32%)	5 (46%)
Male	15 (68%)	6 (55%)
Ethnicity^a^		
NZ European	11 (38%)	7 (67%)
Latin American	4 (14%)	3 (27%)
Māori	2 (7%)	1 (9%)
Tongan	2 (7%)	1 (9%)
Samoan	3 (10%)	1 (9%)
Pakistani	1 (4%)	1 (9%)
Niuean	1 (4%)	1 (9%)
Indian	4 (14%)	1 (9%)
Asian	1 (4%)	0 (0%)
Child’s age, months: mean (SD)	34.7 (9)	31.3 (8)
Diagnoses		
Autism, speech or language delay	15 (63%)	6 (55%)
Global developmental delay	3 (13%)	1 (9%)
None – signs only	6 (25%)	4 (36%)
Diagnosis age, months: mean (SD)	32.6 (8)	31.4 (8.0)
Language level		
Minimal spoken language	21 (95%)	10 (91%)
Fluent	1 (5%)	1 (9%)
Languages spoken at home^b^		
English	20 (59%)	9 (82%)
Spanish	3 (9%)	2 (18%)
Samoan	2 (6%)	2 (18%)
Hindi	1 (3%)	1 (9%)
Urdu	1 (3%)	1 (9%)
Afrikaans	2 (6%)	1 (9%)
Portuguese	1 (3%)	1 (9%)
Cantonese	1 (3%)	0 (0%)
Malayalam	1 (3%)	0 (0%)
Te Reo	1 (3%)	0 (0%)
Tamil	1 (3%)	0 (0%)

a Some children had more than one ethnicity.

b Some families spoke more than one language at home.

**Table 2.** Additional number, type and monthly number of services accessed by children and parents while receiving parent coaching and direct therapy support.

Additional services	All participants (*n* = 22) *n* (%)/mean (SD)	Participant in interview and quantitative survey (*n* = 11) *n* (%)/mean (SD)	Quantitative survey only (*n* = 11) *n* (%)/mean (SD)
**Number of services accessed**	0.64 (0.93)	1.55 (0.78)	0.25 (0.56)
**Monthly hours of support** ^a^	2.8 (3.82)	3.43 (3.58)	1 (0)
**Type of support**			
Speech-language therapy	6 (27%)	5 (46%)	1 (9%)
Wellington Early Intervention Trust^b^	6 (27%)	5 (46%)	1 (9%)
Music therapy	1 (5%)	1 (9%)	0 (0%)
Ministry of Education	1 (5%)	0 (0%)	1 (9%)
Conductive education	1 (5%)	1 (9%)	0 (0%)
Paediatric Autism Communication Therapy	1 (5%)	1 (9%)	0 (0%)
None	12 (55%)	5 (46%)	7 (64%)

a This is reported only for those participants who were accessing at least one service.

b This involves weekly 2.5-h sessions provided by a multidisciplinary team including a music therapist, a speech language therapist and an occupational therapist (Wellington Early Intervention Trust, n.d.). In these sessions, therapists work directly with the child and also provide support to parents. Families opt out of regular support provided by the Ministry of Education in order to access this service.

### Procedure

Each family received 6 months of support based on the ESDM support (Rogers & Dawson, 2010). ESDM is a naturalistic, play-based therapy designed for children who are, or may be, autistic under 5 years old. It can be delivered directly by clinicians, or parents can be coached in this approach. ESDM involves 13 fidelity items for direct implementation with the child (see Supplementary Table 2; Rogers & Dawson, 2010), and another 13 fidelity items guide ESDM parent coaching (see Supplementary Table 3; Rogers & Dawson, 2010). The first 3 months of support consisted of twice weekly 1-h direct support sessions and a once weekly 1-h parent coaching session. In the final 3 months, the amount of direct support remained the same, but the parent coaching sessions were reduced to 1 h fortnightly. The mean percentage of sessions attended across participating families was 78% (range = 55%–89%).

After completion of the support period, parents completed a follow-up questionnaire that included a question about their preference for direct therapy or parent coaching. Those who consented to the interview were given the option to be interviewed in person, over Zoom or on a phone call at their convenience. One participant chose to have the interview over Zoom, and 10 chose a phone call. Parents completed the interview between 1 week and 12 months after completing the low-intensity support. The interviews were audio-recorded, transcribed and uploaded to NVivo. After completing the interview, families received a $20 supermarket voucher to acknowledge their time. The same researcher conducted all the interviews to ensure consistency. Eleven thorough interviews were conducted to provide the rich qualitative responses required for thematic analysis (Braun & Clarke, 2006). The interviews were conducted between October 2022 and September 2023 with a median time of 16 min (range = 11–26 min). Parents were asked to set aside 30 min for this interview. Interviews were conducted by the lead author who was supported to understand and implement the interview guide by members of the team with considerable experience conducting interviews for qualitative research (J.T. and C.W.-W.).

### Materials

Due to the exploratory nature of this research and the scarcity of existing literature related to the research question, the research team developed a questionnaire and an interview guide for this research. The questionnaire was entitled Parent Perceptions of ESDM Coaching and Therapy, and it contained six questions about parents’ perceptions of the support approach. The only question relevant to this current study was, ‘If you had to choose, did you prefer the parent coaching that took place in your home, or the therapy that took place in the Petone Autism Resource Centre?’. Parents were required to select either ‘parent coaching’ or ‘direct therapy’.

The interview guide contained six general topics: (1) overall experience, (2) relationships, (3) enjoyment, (4) outcomes, (5) logistics and implementation and (6) recommendations for other parents (see Supplementary Table 4). Six specific questions fit within these general topics as well as general prompts. Using general topics as a guide rather than specific questions allowed for flexibility during the interview and openness to emerging ideas that were not anticipated prior to the interview (Adams, 2015).

### Data analysis

Parent responses to the quantitative question regarding their preferences for direct therapy and parent coaching were presented descriptively in terms of the number and percentage of parents who preferred each approach.

The interviews were analysed using template analysis with a critical realist approach. Critical realism is an ontology which posits that though one true reality exists, interpretations of this reality are subjective and are ‘always mediated by socio-cultural meanings’ (Terry et al., 2017, p. 24). This approach was well suited to our analysis given the specific context of New Zealand autism supports. Template analysis is a form of thematic analysis which enables the identification of integrative or overarching themes (Brooks et al., 2015; King & Brooks, 2017). Template analysis is well suited to the current study as the data was gathered in two stages. The interviews were uploaded to NVivo for analysis. Template analysis involves (1) becoming familiar with the data, (2) systematically coding a subset of the data, (3) generating initial themes, (4) creating an initial coding template, (5) applying the coding template to further data and modifying if necessary and (6) applying the coding template to the whole dataset (Brooks et al., 2015).

The first author read through the dataset before coding 5 out of 11 interviews using semantic coding. This generated 6 initial themes, 10 sub-themes and an initial template of 16 codes. Next, the coding template was applied to two more interviews and modified twice; each modification was made with collaboration from the other authors, resulting in a reduction from 16 to 8 codes (see Supplementary Table 5). She applied the coding template to the whole dataset and finalised four themes and three sub-themes (Brooks et al., 2015). The themes were named using quotes from the participants. The quotes chosen as theme names were modified to become grammatically correct without changing their original meaning.

Multiple methods were employed at each stage of data analysis to ensure the trustworthiness and credibility of this process. As recommended by Braun and Clarke (2019), we used a collaborative approach to coding and theme development, with regular discussions and reflection regarding code and theme development among all authors. In addition, the first author kept an audit trail through various iterations of the coding template, engaged in reflexive journaling, including keeping a written record of their thinking and decision-making, used data from multiple sources through the use of both interview and questionnaire data, and ensured thorough documentation of changes to themes including records of meetings with co-authors.

Two authors, J.T. and C.W.-W., are experienced with qualitative methods, including reflexive thematic analysis and template analysis. Based on previous research conducted within this population in New Zealand, the authors expected some preference for direct support; however, they were unsure of the reasons behind the suspected preference (Wallace-Watkin et al., 2020). At the time of the study, the first author was the Project Manager of the broader RCT and the current study; therefore, all participants had previous contact with her.

Prior to coding, each participant was allocated a unique identifier (i.e. a random 3-digit number). All identifying information was removed from the interviews during transcription.

### Community involvement statement

This research was co-designed at all stages by P.J., H.W., and C.W.-W. This included developing the interview guide, modifying the coding template, interpreting the results and writing the final study. The lead author, P.J., is an autistic adult. All authors provide support to autistic children and their families. H.W. and C.W.-W. are related to autistic young people.

## Results

### Template analysis

**Figure 1.**
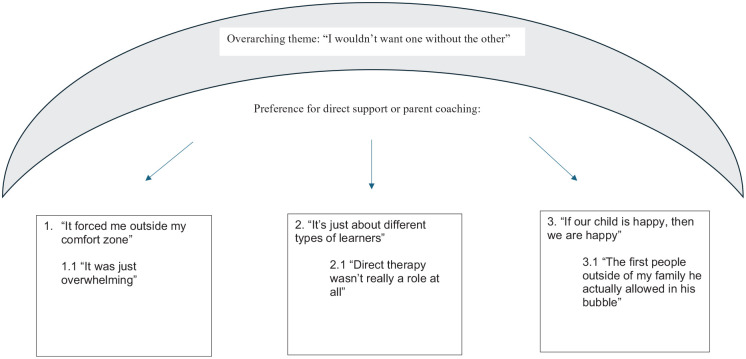
Interconnection of themes.

Four themes from the template analysis provided some explanation for the varying parent perceptions of the direct support compared to the coaching. Themes located using template analysis were ordered hierarchically and included one overarching theme, three top-level themes and three second-level themes. The overarching theme, ‘I wouldn’t want one without the other’, demonstrated that parents wanted access to both supports as they believed they complemented each other. The following three top-level themes show parents’ preferences for either direct support or parent coaching in relation to the different aspects of (1) ‘It forced me outside my comfort zone’, (2) ‘It’s just about different types of learners’ and (3) ‘If our child is happy, then we are happy’. The sub-themes are as follows: (1.1) ‘It was just overwhelming’, (2.1) ‘Direct therapy wasn’t really a role at all’ and (3.1) ‘The first people outside of my family he actually allowed in his bubble’. Parents had varying preferences for being pushed out of their comfort zone, various ways in which they liked to learn, and prioritised their own happiness and well-being as well as that of their child. Aspects of parent coaching and direct support were more or less suited to parents’ varying preferences within these domains. However, overall, parents saw benefits and value in both approaches (see Figure 1).

### Overarching theme: ‘I wouldn’t want one without the other’

All parents agreed that both therapy approaches were beneficial and that they would not want to have one without the other. The parents felt like the supports complemented each other in helping them and their child, ‘I definitely think they’ve been complimentary, I wouldn’t want to have one or the other through these last six months’ (166).

Several parents discussed how the observation in direct support and the discussion and guided practice in parent coaching worked well together. One parent reported that ‘you learn so much watching someone and then one day later you’re turning it around and you’re doing it with someone there to guide you’ (167).

However, many parents reported different preferences in the ratios of parent coaching and direct support sessions. Two parents wanted an equal balance, three wanted more direct support and one wanted more parent coaching. For example, one parent indicated their preferred ratio would be ‘direct coaching 60% and 40% parent coaching’ (192).

### Theme 1: ‘It forced me outside my comfort zone’

Parents’ literal and figurative ‘comfort zones’ influenced their preferences for parent coaching or direct support. In the literal sense, some parents preferred parent coaching because it was conducted in their own homes, which was a comfortable place for them. One parent explained they ‘preferred the home because it was more natural’ (107).

Another parent had to do parent coaching in their in-law’s house as their home was not within the study’s geographical area ‘because I’m based out in [location], they can’t come out that far’ (149). Since they were in someone else’s home, they did not experience the same natural and relaxed feeling as others during parent coaching, ‘I wasn’t quite comfortable, only because it wasn’t in my own home’ (149). This influenced their preferences in favour of direct supports, as they felt more comfortable at the clinic location where they received direct support.

Many parents were figuratively pushed outside their comfort zone as they were taught new techniques and ideas through parent coaching. Some parents felt the push was beneficial because it could ‘elevate’ (167) their play and interactions. One parent stated that they improved because ‘I was being watched and then I was getting direct feedback on that’ (167). Another parent found that the strategies they learned in parent coaching ‘made a really good improvement with my parenting skills’ (149). These parents who enjoyed being pushed outside their comfort zone preferred parent coaching.

Three parents felt uncomfortable being pushed outside of their comfort zone, and they did not feel confident playing and being watched, ‘it’s a bit uncomfortable for me to play with my son and someone is watching me because it is something I don’t think I’m good at’ (177). Therefore, they preferred direct support in the context of playing because it did not require them to play directly with their child.

#### Subtheme 1.1: ‘It was just overwhelming’

Several parents’ preferences and experiences, in particular their experiences in parent coaching, were influenced by their feelings of stress and overwhelm. Some parents felt that parent coaching helped their parenting techniques evolve, making them less stressed. One family reported, ‘We’re a lot more calm. Before, we were just very stressed’ (149). The parent thought their parenting skills had improved due to parent coaching.

However, two parents found the amount and type of information in the parent coaching overwhelming. They reported that the parent coaching was ‘a lot to process in the beginning because it was all new to me’ (149). Another parent reported having to do ‘coursework at night’ (167) because they did not have time for it during the day and would get distracted by other tasks.

Another parent discussed how the environment in parent coaching was stressful as they felt they had to manage their child’s needs and wants while engaging in learning. One parent shared,

The parent coaching was a little bit more burdensome for me because [. . .] it’s kind of challenging in a way that I wasn’t expecting it to be, [. . .] it was easier to go and take [child] to the session than to have like the parent coach come to our house and me have to kind of think about managing [child’s] emotions and needs but also you know tried to be engaged with learning myself. (384)

This parent preferred direct support compared to parent coaching because direct support had fewer components for them to manage and, therefore, was a less stressful environment.

One of the parents had a 2-week break in the middle due to Covid. They found that the break helped them slow down and catch up on coursework as they felt overwhelmed with the continuous work, ‘we were pushing it all the time and I think she just needed some time in the middle to consolidate it. And for us all to take a deep breath and be ready for round two when covid finished’ (167).

### Theme 2: ‘It’s just about different types of learners’

Parents discussed the different ways they and their child learned through direct support and parent coaching, influencing their preferences for either approach. Parent coaching was more explanatory, and direct support was more observational. Overall, the parents emphasised the number of learning opportunities throughout the study regardless of delivery approach:

I could quite often see something in the direct therapy session, and then we might talk about an approach or an idea related to what I had seen a couple of weeks later in parent coaching, like oh that’s what that was, I can see why that’s helpful. (166)

Most of the parents commented on how parent coaching was helpful as it allowed parents to dissect what they had seen in direct support, ask questions and practice the techniques themselves, ‘If it wasn’t for the parent coaching I think it would have been a bit harder understanding the languages they use and the interpretations of, let’s say the ABC’s’ (107).

Many parents voiced that parent coaching helped them become more in tune with their child’s feelings and more aware of their actions as a parent, ‘learning all the techniques I learned in parent coaching helped us understand our son even more and we know when he’s upset and we’ve got a better understanding of how to, not control him, but know when something’s up’ (149).

Parents commented that the learning gained during parent coaching could be implemented even after the programme ended, ‘parent coaching was awesome, I still got all the notes, I laminated them so I can look back on them’ (244). They commented that it had a lasting impact on them and that they were still practicing the techniques they learned:

I understand that it is much better to comment now and I constantly comment now so the learning has stuck with me and when I hear myself asking some questions I’m like ‘Woah, stop’ so you know, it’s made me really conscious of it which is good. (167)

However, while the parent coaching was helpful in aiding the parent’s understanding of the programme content, most parents found it easier to watch the clinician use the techniques with their child in direct support than to use the techniques themselves in parent coaching:

I would say easier watching someone else use the strategies because there’s, I don’t know, a fair amount of double guessing and trial and error and the learning curve with the using the strategies ourselves. (166)

Another parent found direct support full of learning opportunities, similar to parent coaching. This parent discussed how they learned the techniques by watching the direct clinician work with their child:

I was given the option to sit in or not sit in to watch the sessions with [direct clinician], after doing it for six months, I honestly feel like any parent should be sitting in because they just, I think you just learn so much. (701)

In addition, seven parents commented on their preference for direct support regarding their child’s learning opportunities. One parent talked about how their child was ‘learning and developing and being stimulated’ (177) during direct support. They believed that their child made more progress in direct support compared to parent coaching:

I think he, the therapy at the [place] had more results than the parent thing in my case because the therapist made [child] talk for 45 minutes, playing all the time and I don’t have the expertise. (177)

#### Subtheme 2.1: ‘Direct therapy wasn’t really a role at all’

Parents sometimes struggled to learn from and participate in sessions when they were unclear on their role or nature of involvement. This was particularly true of direct therapy. Parents’ preferences were influenced by how much they wanted to be directly involved in playing with their child. Parents had mixed opinions about their involvement in direct support. Some parents enjoyed watching their child in direct support. For other parents, playing did not come naturally, and direct support relieved some of the pressure for them to play with their child, ‘I prefer the therapy at the clinic because I am not a person who likes to play. I don’t like to play. I play because I had to, but it is not something natural in me’ (177). These parents preferred to be less directly involved in playing with their children.

Three parents preferred the direct support sessions because it allowed them to be involved from a distance. They learned by observing and taking notes and photos of the sessions:

In every single session I would take notes so I would also write it down in a book. So I got six months worth of notes [. . .] I asked for her permission if I could take photos as well so she let me do that so I got photos. (244)

Other parents needed clarification about what their role was meant to be in direct support and how they were supposed to be involved. These parents were unsure if they were allowed ‘to step in and go “this is too much”’ (107).

Several parents thought the direct support was ‘boring’ (167) after a few weeks and preferred the parent coaching as it allowed them to be more actively involved in their child’s support, ‘I would say probably the parent coaching aligned more because my preferences is to want to learn and not just be [child’s] taxi driver to therapy sessions but be a therapist at home’ (166).

### Theme 3: ‘If our child is happy, then we are happy’

Parents prioritised their child’s happiness when receiving support and also considered the well-being of the whole family unit. Child and parent enjoyment of sessions contributed to parent preference. For many parents, their experience of ‘enjoyment and comfort in the session is quite closely linked to [child’s] enjoyment, so I’d probably say the therapy sessions were more enjoyable for me in that sense’ (166). Therefore, if their child enjoyed direct support more, the parent enjoyed it more.

Most parents perceived that their child enjoyed direct support more than parent coaching because the child was getting more attention, and the activities were consistently focused on them:

I actually think [child] enjoyed the therapy sessions more, and I attribute that to her getting more one on one attention, during her sessions, which she really enjoys. Whereas there was a fair amount of adult chat [. . .] and the less kind of attention during the parent coaching. (166)

In contrast, two parents reported that their child did not enjoy direct support and that they were not ‘coping with therapy’ (177). These parents talked about their children feeling more comfortable in the parent coaching, potentially due to it being held in their home, which is a natural environment, ‘[child] preferred the home because it was more natural’ (107), and their children showing signs of discomfort and distress in the direct support sessions, ‘[child] not wanting to do it, not wanting to participate [. . .] we had a lot of hard times in the office’ (107).

Some parents discussed an unenjoyable aspect of parent coaching for themselves and their child was the disjointedness of switching between talking to the coach and playing with their child:

the [. . .] interaction between me and [parent coach] and then we’d bring [child] in for a bit and then we kind of distance ourselves to talk a bit and then go back to playing with him and to be honest I feel like that was a little bit like disjointed for him. (384)

These parents preferred direct support over parent coaching as they found it more enjoyable in this aspect.

#### Subtheme 3.1: ‘The first people outside of my family he actually allowed in his bubble’

One determinant of well-being for the child and family was their relationships with both the parent coach and the clinician. Relationships were tied to everyone’s enjoyment of the sessions. Parents and children were reported to have different relationships with their parent coach compared to their direct clinician, which factored into their preferences. Building a rapport with both clinicians was a significant part of creating a positive learning experience for the parents. Parents commented on the benefits of building a rapport with the parent coach and direct clinician:

I had an equal rapport with both I think. I chose to sit in on [child’s] sessions so I think I developed quite a close rapport with [direct clinician] which was really amazing. And then had the connection with [parent coach] [. . .] it was different but equally as great. (701)

The majority of parents reported a deeper relationship with their parent coach compared to the child’s direct clinician. However, many parents saw their children develop ‘a genuine connection’ (167) with their direct clinicians, which helped form a connection between the parent and direct clinician as they shared an interest in the child and the child’s success:

I still felt connected as a parent to the therapists in the ESDM sessions because there was a joint goal of helping [child] and learning to play with [child] in a helpful way, so I would say there’s a connection to both but probably more connected to parent coaching. (166)

Several parents mentioned that it helped with their learning in parent coaching that the parent coaches were ‘patient’ (177), ‘relaxed’ (167) and ‘supportive’ (149). Parents also felt that their parent coaches were flexible depending on their needs, ‘she was taking her time and if I didn’t understand something, she would go back to square one and she wasn’t really fussed about it as long as I understood and was on the right path with it’ (149).

### Quantitative preference data

The quantitative survey results showed that, when asked to choose between approaches, 5 parents (23%) preferred parent coaching and 17 parents (77%) preferred direct support. Of the 11 parents who participated in the interviews, 4 parents (36%) stated a preference for parent coaching and 7 parents (64%) stated a preference for direct support on the survey.

## Discussion

In New Zealand, there is often a reliance on parent coaching to support young autistic children; however, many parents desire increased access to direct support (Wallace-Watkin et al., 2022). This appears to be the first study to examine parents’ preferences for parent coaching and direct support after having received both approaches for their autistic or potentially autistic (‘autistic’) child. The consensus was that parents found both supports to be beneficial and complementary to each other. Only when forced to choose an overall preference were parents more likely to choose direct support. The nuance of the qualitative data showed that parent preferences for direct support appeared to be influenced by several factors, including feeling uncomfortable with the structure of parent coaching and being observed playing, not feeling confident in their ability to implement the techniques, and their child’s perceived enjoyment and increased progress in direct support.

The most common reason parents preferred direct support was that they prioritised their child’s progress and enjoyment in supports. Most parents believed their child had more enjoyment and progress in direct support than when the parents implemented the skills they learned in parent coaching. While research shows that parents have higher levels of confidence in supports when they are delivered by university research programmes, these beliefs may stem from a lack of confidence in their ability to implement the techniques, as mentioned by several parents (Bent et al., 2023). Research shows that parents’ confidence in their ability to deliver support strategies can be either a facilitator or barrier to the support’s success (Carruthers et al., 2022). This suggests that supports could focus on increasing parental confidence in implementing techniques to increase the likelihood of success. Research indicates that parents feel more confident delivering the techniques when they feel supported and empowered by the clinician and believe in the techniques they are delivering (Carruthers et al., 2022; Leadbitter et al., 2020).

Parents of autistic children often report higher stress levels (Estes et al., 2019; Wallace-Watkin et al., 2023). Similar to previous research, some parents indicated that parent coaching reduced stress (van Noorden et al., 2022; Weitlauf et al., 2020; Zhou et al., 2018). However, some parents preferred direct support because it relieved the pressure of having to deliver the support themselves. This relates to findings from a study that looked at the effects of early support on parents of autistic children that indicated parents are less strained when their child receives support from a clinician rather than from themselves (Estes et al., 2019). A few parents preferred direct support because they felt overwhelmed by the amount of information delivered in parent coaching. They found balancing life while completing parent coaching challenging. Evidence suggests that parents need breaks or additional parent-focused support to give them time to recharge and complete life tasks (Galpin et al., 2018; Jurek et al., 2022; Whitmore, 2016).

Some families were uncomfortable during parent coaching due to its structure. Two families were uncomfortable playing with their child and being observed. These families were from Latin American and African backgrounds, suggesting a possible cultural influence. While little is known about the views of those from these backgrounds towards play, this potential cultural influence aligns with research from Sengupta et al. (2020), who identified that for many Indian parents, playing with their children was challenging and did not align with their cultural values. The low-intensity support in the current study was developed in the United States and may include Western assumptions, such as the underlying beliefs regarding parent–child play. As such, it may not be equally suited to individuals of all cultures.

There were several drawbacks to the delivery of direct support in a clinical setting, leading to many parents preferring the home-based nature of parent coaching. Parents felt comfortable and relaxed in their natural home environment compared to attending the clinic for direct support. Some parents discussed challenges in transporting their child to the clinic, such as whether their child wanted to stay in the house and factoring in the transportation time from their home to the clinic. Parents in New Zealand have identified transportation to access services as a significant barrier (Wallace-Watkin et al., 2023). Another challenge raised related to children not settling into the clinic environment and parents being uncertain how or when they could intervene when their child was upset. Whereas, at home, parents felt more comfortable calming their children, and their children were generally more settled.

In low-resourced areas, such as New Zealand, there is often just one service, in this case parent coaching, provided to families of autistic children (Kasilingam et al., 2019; Wallace-Watkin et al., 2022). For this reason, we used a quantitative survey question to understand parents’ preference for parent coaching or direct support *if they were forced to choose* between options. However, best practice may involve supporting autistic children and their families using a variety of relevant and preferred delivery methods (Trembath et al., 2022). As such, parents were able to express more nuance and variation in preferences within the qualitative interviews. The low level of support in New Zealand was reflected in the additional services accessed by families across the duration of the study. Families only received an average of two additional types of support for 1½ h per week. As such, the parent coaching and direct support provided within this study were the main services they received and likely the primary influences on their preferences. Interestingly, many families had chosen to opt out of standard services provided by New Zealand’s Ministry of Education, which predominantly employs a consultation approach, in order to access a service with a greater direct delivery component. This lends further evidence to parents’ desire for direct support for their autistic child.

The main implication of this study is that parents consider parent coaching and direct support complementary to each other, and practitioners should consider providing both approaches together rather than exclusively one approach. Where possible, conducting parent coaching without the child present may allow parents to focus solely on their learning without splitting their attention between the coach and their child. In addition, it would remove the disjointedness of the parent switching between talking to the coach and playing with their child. Parents may benefit from the implementation of strategies aimed to help increase parent confidence and reduce stress reduction (Prata et al., 2018). Finally, home-based delivery may reduce geographic barriers and help children and parents feel more comfortable (Wallace-Watkin et al., 2023).

This study adds to the sparse amount of literature on understanding parents’ preferences for different support approaches; however, it is not without limitations. Out of the 22 eligible parents, only 11 showed interest and consented to participate in the interviews. This selective participation may have introduced bias to the interviews, as the findings may predominantly reflect the perspectives of those who found the programme enjoyable or beneficial and also those participants who were more proactive in seeking out additional support. Consequently, the views of parents who disliked the programme and did not find it suitable and those who struggle to navigate services may be underrepresented in the interview. In addition, although the parents were deemed to speak sufficient English to participate in the coaching, many did not speak English as a first language, which could have reduced the length of the interviews, which were conducted in English, and limited the depth of their responses. Furthermore, in many cases, the interviews were completed as part of a large battery of assessments for the broader RCT and parents were told that it would take up to 30 min. It is possible that the number of assessments placed burden on families and reduced the length of time compared to doing the interviews in isolation. Despite these limitations, the interviews still led to a rich analysis and the development of deep themes. The interviews were only conducted with the parent who was the primary participant in the broader study, so little is known about the preferences and perceptions of other family members. The sample was small, with only two fathers represented. Research shows that father–child relationships differ from mother–child relationships (Cabrera, 2019; Liu et al., 2021). Therefore, if more fathers had participated in the interviews, different themes and preferences may have come to light. Additional participant characteristics such as employment or education level may have influenced their responses and the results of the study, as 55% had a university qualification and 2% were employed full-time. Furthermore, the parents interviewed finished the study at different times. Those who finished the study over 6 months ago may have more varied and less specific memories compared to those who completed the study more recently. This may be why some parents’ preference in the survey immediately after receiving the support differed from some of their qualitative statements. This may also have been because the survey offered a forced choice between parent coaching and direct support, while the interview allowed for more flexible parent responses.

The results from this study indicate several directions for future research, including the evaluation of parent–child interaction preferences in non-Western cultures and how supports could be adapted to accommodate these preferences. Furthermore, in some countries other than New Zealand, such as the United States, there is a greater focus on direct support and access to parent coaching may be more limited (Monz et al., 2019). Consequently, it is essential for researchers to investigate delivery preferences across different geographical and cultural contexts to understand the broader demand for direct services compared to parent coaching and to tailor supports accordingly. This should include consideration of families opting out of publicly funded coaching services. This cross-cultural examination will provide valuable insights into how supports can be optimised to meet the diverse needs of families worldwide. It would also be of interest for researchers to analyse how the services that a family is currently accessing influence their preferences for future services. Researchers could also evaluate the feasibility and effectiveness of group parent coaching sessions in increasing parent confidence, perceived child enjoyment and decreasing parent stress. Third, future research could investigate the possibility of adding planned breaks into the provision of support. These breaks may help reduce stress and burnout and increase performance and enjoyment. Finally, this research into parent preferences could be extended into parents’ preferences for other delivery approaches, such as a comparison of preferences for parent coaching or teacher coaching.

## Supplemental Material

sj-docx-1-aut-10.1177_13623613241287300 – Supplemental material for ‘I wouldn’t want one or the other’: Understanding parents’ preferences for direct support or parent coaching for young autistic children Supplemental material, sj-docx-1-aut-10.1177_13623613241287300 for ‘I wouldn’t want one or the other’: Understanding parents’ preferences for direct support or parent coaching for young autistic children by Phoebe Jordan, Carla Wallace-Watkin, Jessica Tupou, Sarah Pillar and Hannah Waddington in Autism
