# Supplementary material for: ‘I wouldn’t want one or the other’: Understanding parents’ preferences for direct support or parent coaching for young autistic children
Source: Autism. 2024 Oct 12;29(3):740–53. doi: 10.1177/13623613241287300 (PMC11894850; doi:10.1177/13623613241287300)
Supplement: sj-docx-1-aut-10.1177_13623613241287300 – Supplemental material for ‘I wouldn’t want one or the other’: Understanding parents’ preferences for direct support or parent coaching for young autistic children [file sj-docx-1-aut-10.1177_13623613241287300.docx]

**Supplementary Table 1.**

*Description of the Early Start Denver Model fidelity items.*

| **Fidelity items** | **Description** |
| --- | --- |
| 1. Managing child attention | This item targets the child’s visual and auditory attention to the adult and to the materials; that is, the ability of the adult to get the child’s attention on a teaching activity and then “step into the spotlight”—the center of the child’s visual attention, so that the child attends to the adult. |
| 1. ABCs | This item rates the clarity of the teaching interactions, frequency of teaching interactions, and the appropriate use of repetitions during the activity. |
| 1. Instructional techniques | Efficacious application of instructional techniques: Did the therapist use shaping, fading, prompting, and/or chaining techniques appropriately, and was error correction managed well to elicit and teach new behaviors? |
| 1. Child affect and arousal | Use this item to address adult management of child emotional state, or activity level: such characteristics as a tired, lethargic, or underaroused child, a passive, perhaps avoidant child, a child who is whining, escaping, frustrated, distressed, upset with someone’s coming and going, upset because a favorite toy was put way, or an overactive, high-energy child who is not settling into an activity. This is not about overt behavior problems—those get coded in a different item. This is about optimizing a child’s mood, state, or activity level for participation in learning. |
| 1. Unwanted behaviours | When a problem behavior occurs, does the therapist seek or demonstrate a clear understanding of the function of behavior and use appropriate techniques to elicit more appropriate behavior? Unwanted behaviors involve aggressive acts to others, self-injury, crying, marked fussing or screaming, significant stereotypies, throwing or destroying materials, and active, oppositional refusal to follow instructions. Lack of cooperation, poor attention, overactive avoidance, whining, and passivity would not be coded. |
| 1. Dyadic engagement | This involves a type of social engagement in which adults and children are acting in a coordinated fashion. At its best, the child is aware of the adult’s activities and the adult is an interactive partner, and the child demonstrates this through shared gaze, directed, intentional communicative exchanges, and smiles. Both partners lead, and both follow. In a more structured, material-based activity, dyadic exchanges may not occur throughout the activity, but rather in moments of socially engaged, enjoyable, reciprocal acts. These are expected to occur somewhere in every teaching episode. |
| 1. Child motivation | This item refers to the child’s motivation to perform this specific teaching task multiple times, through the number of trials the adult requests. If problems of motivation in this episode lead to problems with state or unwanted behaviors, then all the appropriate items would be coded. Child choices are a very important aspect of this item. In a naturalistic teaching episode, this involves child choice of materials/activity. In an activity that does not involve objects (songs, play) the adult may “offer” the activity, but adult still follows the child’s lead in determining whether to continue. In an adult-directed teaching episode, this can include child choice of reinforcers, or child choice of a preferred activity in which a didactic teaching episode will be embedded. Valuing child choice does not preclude modeling a new toy or activity, or taking a child through an activity for the first time to introduce it, even if the child is mildly protesting. However, it does preclude continuing an activity in the face of marked child protest or disinterest unless the activity is necessary for child safety, hygiene, and so on. An adult who is consistently suggesting a new activity to the child - “Let’s play food, okay?” is not giving adequate opportunity for choices. This is not a problem when done once in a while, but if it occurs more often than that then the adult is being too directive and not creating and following child choices and leads. |
| 1. Adult positive affect | The adult displays rich, genuine, and natural positive affect throughout the episode matched by child positive affect. Positive affect permeates the episode, is well matched to the child’s needs and capacities, does not overarouse the child, and serves teaching well. |
| 1. Sensitivity and responsivity | This refers to adult’s attunement to child states, motives, and feelings. A sensitive and responsive adult acknowledges communicative cues, whether verbal or gestural, by verbalizing or by acting contingently according to the child’s communication so that the child seems to have been “heard.” Or, in the face of an affective cue, the adult responds empathically to the child’s emotional state by mirroring the emotion and communicating an understanding of it. The adult does not reinforce unwanted behavior, but acknowledges the child’s cues and responds appropriately given the situation. The adult uses a range of techniques including modeling, restatement, expansion of child utterances, and repetition of child utterances embedded in meaningful activities. |
| 1. Communicative opportunities | This item addresses the number of pragmatic functions expressed in child communications and elicited by the adult. Examples include requesting, commenting, naming, protesting/affirming, seeking help, being “all done,” greeting, or imitating the adult’s sounds or gestures with eye contact. Children’s imitation of an adult action on an object, without accompanying gaze, vocalization, gesture, and so on is not considered a communication for this item. |
| 1. Adult language | Is the therapist’s language appropriate for expanding the child’s language level in terms of vocabulary, syntax, and pragmatics? This includes comments to the child, language models, and appropriate narration of the actions or themes involved in the activity. |
| 1. Structure and elaboration | Does the therapist develop a four-part joint activity: (1) a set-up in which child chooses activity and helps adult set up the theme; (2) a middle in which both participate equally, building, and co-constructing the theme; (3) elaboration to encourage flexible, varied use of actions and materials by using multiple materials and varied schemas, or through theme and variation; and (4) an ending in which the timing to close down the activity was fitting and the child is well supported through the transition to the next activity? Does the adult target multiple objectives from different developmental domains? |
| 1. Transitions | Does the adult skillfully transition between activities or locations to maximize child attention, motivation, and independent physical transition to new activity? |

**Supplementary Table 2.**

*Description of the Early Start Denver Model parent coaching fidelity items.*

| **Fidelity items** | **Description** |
| --- | --- |
| 1. Greeting and checking in | The coach greets parent(s) and child. The parent(s) provides a snapshot of the week and identifies their focus with child for the past week, usually set at the previous session. Therapist gathers any data that parent(s) has completed since previous session. Parent(s) may raise other questions or concerns, which the therapist acknowledge. This is a brief period that sets a base for quickly moving into action. |
| 1. “Warm up” parent-child joint activity | This phase allows the coach to observe and evaluate parent(s)-child progress based upon what was discussed and practiced in the last session. (1.) The focus of the interaction emerges from the parent(s)’ sharing of the week’s activities and theme. (2) An uninterrupted parent-child activity occurred that allows parent(s) and child to move into action and allows the coach to gauge progress. (3) After the activity, the coach invites the parent(s) to reflect on the activity, to evaluate the extent to which the parent(s) goals were realised. (4) The coach then comments constructively in a way that reinforces parental use of learned techniques. (5) there is a smooth transition into the next phase. |
| 1. Introduction of the topic of the day | This phase sets the main topic for the session and mentions the sequence of events that will happen in the session (if this has not occurred earlier). The coach: (1) defines the topic from the manual; its goal, main parenting strategies, and effects on child behaviour adjusted well for time; (2) ties the topic to parent(s) goals for the child; (3) ties the new topic into previous observations pr discussions with the parent(s); (4) applies multimodal strategies (print materials, verbal explanation, visual demonstration) that best fit the parent(s)’ learning needs; (5) solicits parent(s) input to assess understanding; and (6) demonstrates sensitivity to parent(s)’ cultural and child-rearing beliefs. |
| 1. Coaching on the week’s topic | This phase focuses on the coach’s skills to support [aren’t attention and success at using the topic skill. Coaching does not interfere with the ongoing activity, improves parent(s)’ practice as activity continues or from one activity to the next, and occurs across at least two activities during the session. By the end of the coaching, the parent(s) can state the main strategies and child goals to practice before the next session. Coaching strategies involve: (1) tying the key teaching strategies to parent actions and child behaviour and goals; (2) provision of enough support for the parent(s) to reflect on experience and evaluate key interactions using open-ended questions and active listening; (4) another practice interaction using a different activity, with enough support for success; (5) coaching practice (e.g., active listening, providing feedback, direct modelling) fit parent(s)’ learning styles, understanding level, values. |
| 1. Coaching on the week’s topic | This is the same as above as it is coded twice, once for each activity. |
| 1. Closing | In the last 10 minutes or so of the session, the coach and parent(s) carry out a balanced discussion in which the coach: (1) makes time for any topics the parent(s) brought up for discussion earlier and addresses them; (2) elicits the parent(s)’ understanding of the topic, the parent(s) and child skills involved; (3) supports parent(s) to consider use of the techniques in multiple activities and contexts of daily life; (4) invites parent(s) reflection and evaluation of the utility of the session activities; (5) reviews the next meeting time and (6) says goodbye to both parent(s) and child in a smooth transition out of the door. Parent(s) and child leave on a positive note. |
| 1. Collaborative | The coach works with the parent(s) as a partner to reach common goals rather than deciding on the goal for the parent(s) to achieve or leaving all responsibility to the parent(s). It’s about the balance in the relationship. |
| 1. Reflective | Throughout the session the coach reflects on (1) what they have observed, (2) the relationship between the learning goals and observed actions, (3) effects of parent behaviour on child behaviour and (4) emotional expressions that are occurring. These reflections proceed and lead to evaluations, ideas and suggestions, so that the coach’s line of thinking is transparent to the parent(s). The coach invites parallel reflections from the parent(s), and supports the parent(s) to use reflection to lead to evaluation and action plan. Thee reflections focus on child goals, emotions, motivations and communications and the relations among context, parent(s) behaviour and child behaviour. |
| 1. Non-judgemental | The coach uses descriptive rather than evaluative or judgemental language to point out relations between parent actions and child behaviour. |
| 1. Conversational and reciprocal | The interactions between the coach and therapist are balanced. The tone is conversational rather than didactic. The therapist listens well and responds to parent(s)’ topics. The tone is friendly, warm, encouraging and optimistic and maximises child and parent enjoyment and success. |
| 1. Ethical conduct | The coach demonstrates appropriate professional behaviour and does not talk about other patients or own children, criticising other therapists or approaches, too much self-disclosure, discouraging comments, jokes, crossing of professional boundaries, making a request for therapist benefit, coercing child or family. |
| 1. Organising and management of session | Data, materials and furniture, readiness, child is occupied, physical space in well organised, session follows protocol, space and structure fit family and child well, transitions smooth. |
| 1. Managing parental implementation difficulties | Parent implementation difficulties often reveal themselves in (1) lack of follow through in interventions the parent(s) has themselves requested e.g., homework, sleep, eating, etc; (2) habitual lateness, cancellation, or no show; (3) a great deal of discussion and explanations that interfere with time spent working with child; (4) many excuses for child behaviour e.g., tired, ill, etc; (5) philosophies of parenting that are fundamentally contrary to the intervention (not wanting to reward child for appropriate behaviour). |

**Supplementary Table 3.**

*Interview guide*

| **Topics** | **Possible Questions** | **Probes** |
| --- | --- | --- |
| Overall experience. | Tell me about your experience with the ESDM parent coaching and direct therapy. | - *General ice breaker, no probes needed.* |
| Relationships | How did parent coaching and direct therapy compare with relationships? | - *In your relationships with the therapists? (feel supported?)* - *In your relationship with your child?* |
| Enjoyment | How did parent coaching and direct therapy compare with the enjoyment of sessions? | - *In your child’s level of enjoyment and comfort?* - *In your level of enjoyment and comfort?* - *In comfortably aligning with your values and preferences? (e.g., parenting styles aligning with coaching/therapy techniques?)* |
| Outcomes | How did parent coaching and direct therapy compare in outcomes? | - *In aiding your understanding of what was happening in therapy/ESDM?* - *In meeting your child’s needs and goals? (child outcomes)* - *In meeting your own needs and goals? (whānau outcomes)* |
| Logistics and implementation | How did parent coaching and direct therapy compare in logistics and implementation? | - *Time commitments* - *Location* - *Workload* - *The person using the strategies, (was it easier to use them yourself or to watch the therapist use them?)* |
| Recommendations for other parents. | Would you recommend parent coaching or direct therapy to your friends and family? And why? |  |

**Supplementary Table 4.**

*Template 4; 10/5/23*

| **Name** | **Description** |
| --- | --- |
| 1. **“I wouldn’t want one without the other”** | Parents thought both supports were beneficial, and the supports complimented each other. However, many parents talked about preferring the supports in different ratios. |
| 1. **“It forced me outside my comfort zone”** | Parents experienced being pushed out of their comfort zone. This was viewed both positively and negatively. Some parents preferred parent coaching because they felt it positively pushed them outside of their comfort zone. Some parents preferred direct therapy because they did not want to be pushed out of their comfort zone. |
| 1. *“It was just overwhelming”* | Some parents felt that parent coaching helped their parenting techniques evolve and therefore they felt less stressed. Whereas other parents felt that the information they were given in parent coaching was overwhelming and created stress for them. Some parents did not feel comfortable playing with their child or confident in implementing ESDM techniques during parent coaching. They preferred direct support as it removed that stress for them. |
| 1. **“It’s just about different types of learners”** | Parents learned so much and emphasized the amount of learning opportunities throughout the study. They reflected on things that helped them learn such as supportive coaches. Parent coaching and direct support helped the parents learn in different ways. Parent coaching was more explanatory and direct support was more observational. Most parents said they learned more in parent coaching compared to direct support. However, it was easier to watch someone else use the therapy techniques (direct support) than to use them themselves (parent coaching).  Many parents preferred direct support for their child’s learning, as they felt it was more beneficial for their child rather than their child receiving ESDM from them. |
| - 1. *“Direct therapy wasn’t really a role at all”* | Parents had positive and negative views of their involvement in their child’s therapy. Some parents did not know what their role was meant to be in direct support and how they were supposed to be involved. Parents were less involved in direct support and more involved in parent coaching, so the parent coaching was more enjoyable for them. Other parents did not enjoy playing and implementing the ESDM techniques, therefore they preferred direct support as did not have to be involved in it. |
| 1. **“If our child is happy, then we are happy”** | Parents’ experience of enjoyment in support was closely linked to their child’s enjoyment in the therapy. Most children enjoyed direct support more therefore parents enjoyed direct support more regardless of whether they were involved in it. Parents perceived that their child preferred to be involved in direct support because it was more fun for them. |
| - 1. *“The first people outside of my family he actually allowed in his bubble”* | The parents had different kinds of relationships with the parent coach compared to the direct therapist. Parents found that they had strong relationships with the parent coach compared to their children who usually had a stronger relationship with the direct therapist. However, the parent and the direct therapist were able to connect through their shared interest in the child’s success. Parents discussed the social aspect of support. Parents got to see different sides of their children as they interacted with those outside of the immediate family. |
